# Effect of simple interventions on the performance of a miniature MFC fed with fresh urine

**DOI:** 10.1016/j.ijhydene.2021.07.171

**Published:** 2021-09-28

**Authors:** Asimina Tremouli, John Greenman, Ioannis Ieropoulos

**Affiliations:** Bristol BioEnergy Centre, BRL, University of the West of England, T-Building, Frenchay Campus, Bristol BS16 1QY, UK

**Keywords:** Microbial fuel cells design, Bioenergy, Ceramic membranes, Urine

## Abstract

The aim of the present study is to enhance the performance of a microbial fuel cell (MFC) design by making simple interventions. Specifically, terracotta “t” and mullite “m” ceramics are tested as membranes while carbon veil and carbon cloth are used as electrodes. In the case of “m” cylinders different dimensions are examined (m: ID 30 mm x height 11.5 mm; sm: ID 18 mm x height 18 mm). The units operated continuously with urine as the feedstock. The best performing is the sm type (60–100 μW), followed by the t type (40–80 μW) and the m type (20–40 μW). Polarisation experiments indicated that activated carbon on the anode enhances the power output (t: 423 μW, sm: 288 μW). Similarly, the increase of the surface area and the addition of stainless steel mesh on the cathode improves the power performance for the “sm” and the “t” units. Furthermore, it is shown that the design with the smaller internal diameter, performs better and is more stable through time.

## Highlights

• A microbial fuel cell design is optimized by making simple modifications.
• The design performs better when its internal diameter is decreased from 30 mm to 18 mm.
• Activated carbon on the anode drastically increases the maximum power output.
• The increase of the cathodic surface area boosts the power output.
• Stainless steel mesh on the carbon cathode electrode enhances the power output.

## Graphical abstract

**Figure:**
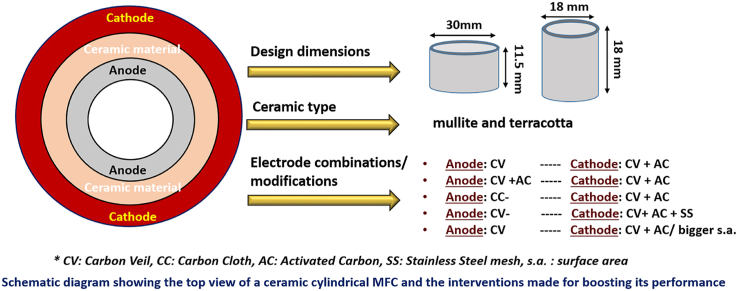

## Introduction

During the last twenty years, many microbial fuels cell (MFC) systems have been developed at different research laboratories. The unique feature of the technology to directly convert the chemical energy of organic compounds to electricity using bacteria as catalyst is attractive for many researchers [1,2]. Several designs have been reported, using different construction materials when fed with various types of biodegradable substrates, under the scope of optimizing the system performance for different applications [[3], [4], [5]].

The MFC design is a crucial factor that affects the technology performance. Single chamber configurations produce higher power densities in comparison with the dual chamber MFCs [6,7].Thus, in order to minimise the high internal resistance which drastically contributes to the low power output values, many designs and electrode materials have been proposed [[8], [9], [10], [11], [12]]. Further to the reactors design, the configuration of the electrodes and the electrode materials influence to a great extent the performance of an MFC. In particular, the electrodes should combine several characteristics, for boosting the power density of the system. Specifically, the desired traits of the electrodes are the high effective surface area, the high electrical conductivity, their stability and durability, their biocompatible properties (for biotic electrodes), the low pore clogging as well as their low cost [13]. In this direction, carbon electrodes and metal electrodes have been extensively tested both as anode and cathode electrodes [[14], [15], [16], [17]]. In an effort to enhance electrodes efficiency, researchers have also tried to modify the above materials or build new 3D structures with improved characteristics (e.g. 3D carbonaceous electrodes) [[18], [19], [20], [21], [22]]. For example Lai et al. [21] studied the zinc- (FZ) and nickel-coated (FN) 3D anodes on the performance of a dual chamber MFC. A power density of 142.4 mW/m^2^ and 138.6 mW/m^2^, was achieved for the FZ electrodes and the FN electrodes, respectively. Moreover, Singh and Verma [22] fabricated nickel (Ni) nanoparticles- (NPs) dispersed web of carbon micro-nanofibers (ACFs/CNFs) and used them as the electrodes in a dual chamber MFC achieving power density 1145 ± 20 mW/m^2^. The above approaches could replace expensive electrodes used in MFCs.

Moreover, the selection of the proper separator is another challenge to overcome for implementing the technology. Typical separators (e.g ion exchange membranes), hinder the real application of the MFCs because of their high cost and their high contribution to the internal resistance of the cells [23]. In order to overcome this issue, alternative separator materials (e.g. porous fabrics, ceramic membranes, glass fibers, J-cloth, zirfon) have been suggested [[24], [25], [26], [27]]. For example, Das et al. [28]

Reported that the Nafion-alternative membrane using poly (vinyl alcohol) (PVA) crosslinked with glutaraldehyde (GA) produced higher power density (158.28 mW/m^2^) than those typically reported for domestic wastewater fed MFCs. Moreover, the utilization of the ceramic material is a promising option since clay is abundant in the environment and thus it is low cost. Additionally, due to its robustness it can be simultaneously used as a membrane and as a structural element, thus reducing the overall construction cost of the system [23,29,30]. In this view, Raychaudhury and Behera [31],

Fabricated ceramic membranes by blending rice husk ash (RHA) with soil. They observed that the addition of RHA improved the maximum volumetric power of the MFC by approximately 61% (2.14 W/m^3^).

The MFC technology can treat many types of wastes originating from different sources such as industry, livestock farming, domestic wastewater, neat urine and food waste [9,[32], [33], [34]]. However, for high strength or heavily polluted effluents, although the organic removal is satisfying when used as feedstocks in MFCs, the energy output is very low [35]. Further to the toxic elements that might be present, these complex substrates cannot be directly consumed from electrogenic bacteria. Consequently, the organic matter is removed from antagonistic microbes existing in the anode chamber [36]. Athough some high strength and heavily polluted waste streams need pretreatment before they are polished with the MFC technology, neat urine is an ideal substrate for MFCs. Urine is highly conductive, has buffering capacity and also has a big portion of biodegradable organic matter. The above traits along with its abundance, makes it a suitable feedstock for the technology and the development of decentralized treatment processes [37,38].

This work is an effort to combine the aforementioned aspects that move the MFC technology towards its practical implementation. In this respect, a urine-fed single MFC design was assessed [9]. At the same time, the improvement of electricity production from relatively simple and low cost modifications to the anode and the cathode electrodes was tested. Specifically, the effect of cathode surface area, the addition of stainless steel in the cathode as well as the addition of activated carbon on the anode, were demonstrated. Moreover, it was evaluated the effect of different ceramic types and dimensions of a newly MFC design.

## Materials and methods

### Design and operation

**Fig. 1:**
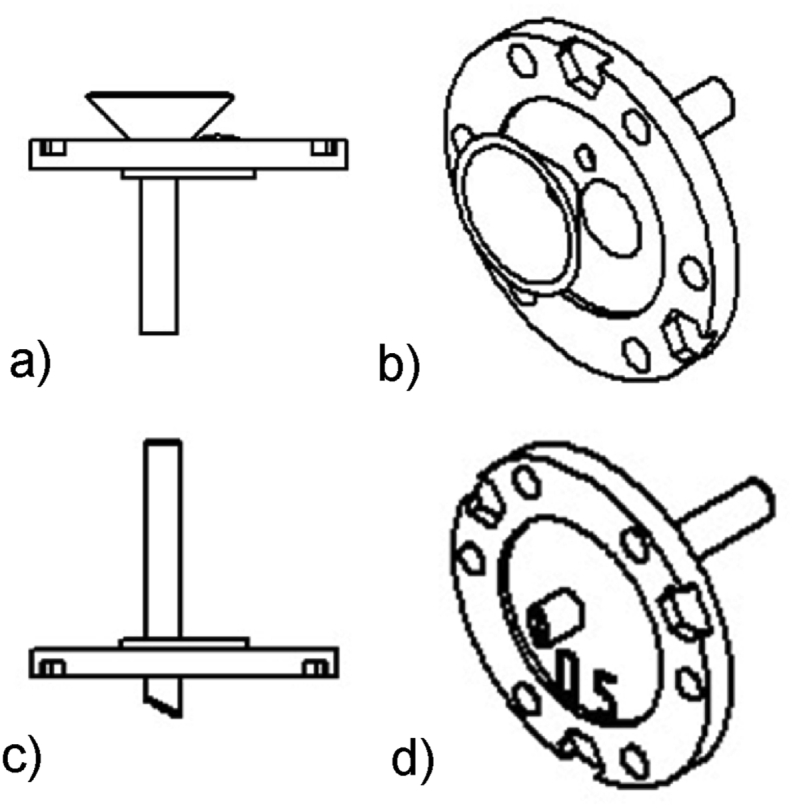
Schematic diagrams from different views of the a), b) top lids and c), d) bottom lids.

Twelve sets of different ceramic MFCs (4 ml volume) in triplicates were tested. The 36 MFC units were constructed using the same design concept as previously described [9]. Specifically, ceramic tubes open at both ends were used as the separator. Two 3D printed acrylo-nitrile butadiene styrene (ABS) lids were used for sealing the tubes and for supporting the inlet and outlet tubes. In order to avoid leakages during the substate flow, a funnel was incorporated on the top lid and on the inlet tube. The effluent overflew from the constant–level outlet tube placed near the top of the cell so that the units can operate in continuous mode. Moreover, the positioning of the tubes ensures that the fuel is gradually driven from the base of the unit to the top surface of the anolyte, thus increasing the time of the new inlet within the reactor. The outlet is placed inside the anode chamber for gaining space under the view of stacking multiple units together. (Fig. 1).

**Fig. 2:**
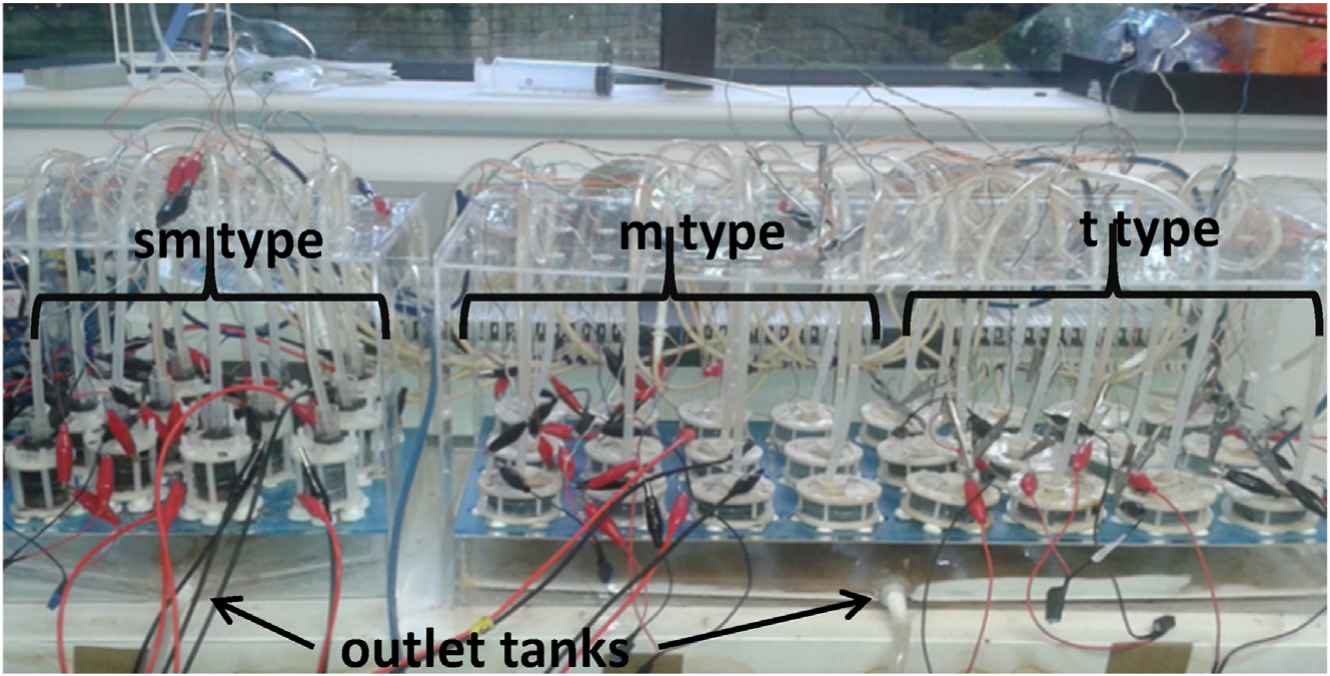
The experimental set-up of the 36 cells.

The cathode electrodes were open to air and they consisted from carbon veil sheet coated by activated carbon (AC) [9]. Stainless steel was wrapped around the cathode electrode reassuring both a better attachment of the cathode electrode on the ceramic tube and a connection point for the crocodile clips. Fig. 2 shows the experimental set up of the 36 MFC units.

Under the scope of enhancing the performance, different materials were modified and tested. Specifically, plain carbon veil and plain carbon cloth were investigated as anode electrodes, while the addition of activated carbon (AC) on their surface was examined [9].The AC anode electrodes were prepared following the same procedure for cathodes, as previously described. Different surface areas (s.a.) of the cathode electrodes were examined (s.a.:13.75 cm^2^ and s.a.: 22.86 cm^2^); the effect of the addition of stainless steel mesh (SS) on the cathode electrode was also tested. Specifically, two types of ceramics were used, mullite and terracotta. Terracotta or “t” cylinders (outer diameter or OD 40 mm x ID 34 mm x height 11 mm) and mullite “m” cylinders (m: OD 40 mm x ID 30 mm x height 11.5 mm; sm: OD 26 mm x ID 18 mm x height 18 mm) had an open porosity of approximately 27% (t, sm) and 6% (m). Table 1 shows the different characteristics of the twelve MFC types tested in triplicates.

**Table 1:** The twelve MFC types tested in triplicates (CV: carbon veil, CC: carbon cloth, AC: activated carbon, SS: stainless steel).

MFC type abbreviation	Anode Composition (s.a 64.80 cm^2^)	Cathode Composition (s.a 13.75 cm^2^)
sm1	CV	CV
sm2	CV with AC	CV
sm3	CC	CV
sm4	CV	CV (s.a 22.86 cm^2^)
m1	CV	CV
m2	CV with AC	CV
m3	CC	CV
m4	CV	CV with SS addition
t1	CV	CV
t2	CV with AC	CV
t3	CC	CV
t4	CV	CV with SS addition

For comparison, the same acclimation procedure and the same operation mode were followed for all units. The inoculation occurred in during three batch mode cycles (under 2 kΩ external resistance per cell) [9]. For the acclimation of the biofilm, 50% of activated sewage sludge supplied from the Wessex Water Scientific Laboratory (Saltford, UK) and 50% of fresh urine was used as the feedstock. Urine was collected daily from adult individuals. When the enrichment of the cells was completed, only urine was added in the anode chamber and the units operated in continuous mode. Additionally, the MFCs were fed individually (Fig. 2). Urine was pumped (205U, Watson Marlow, Falmouth, UK with 36 channels) from a common tank. The cells were fed under a very low flow rate (0.55 ml/h) due to the limited urine availability.

**Fig. 3:**
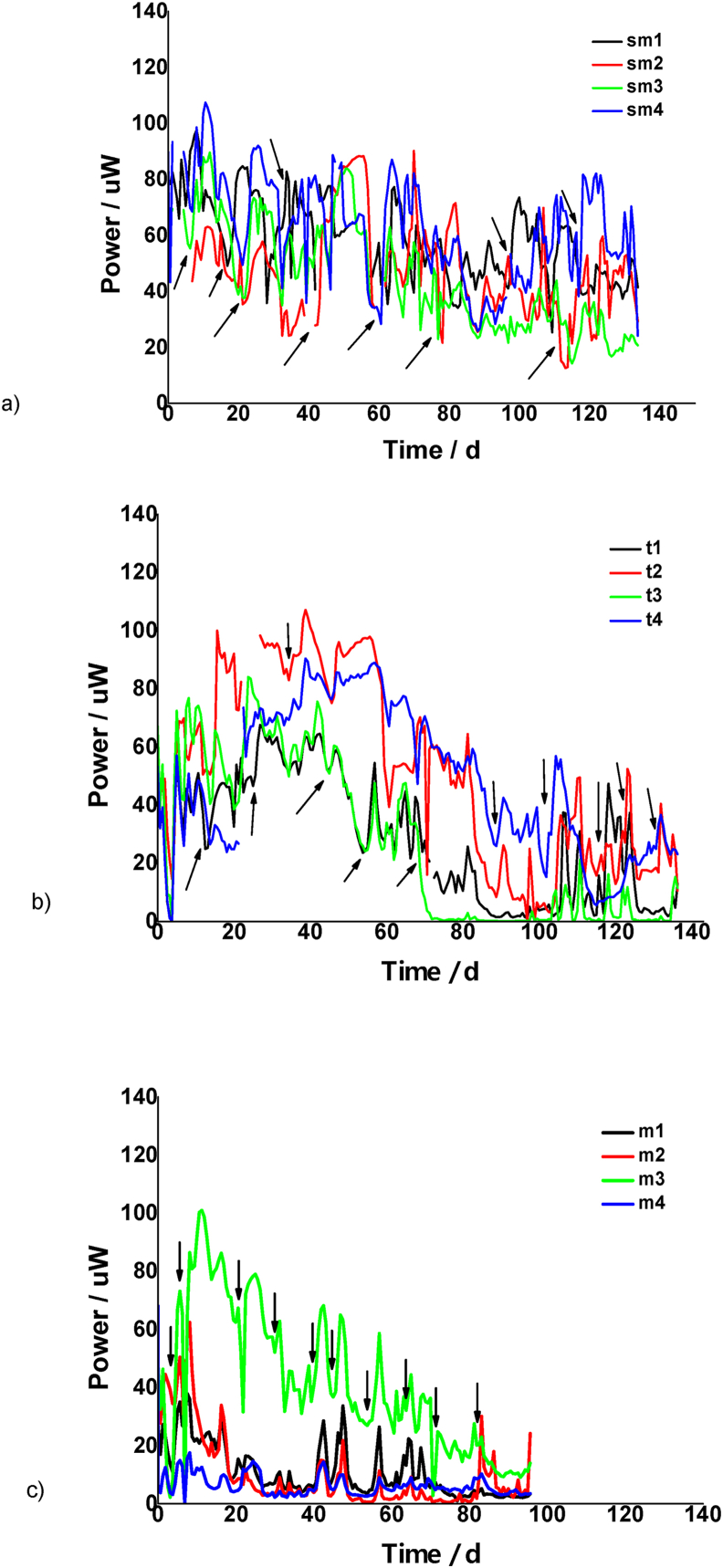
Comparison in the power behavior among the MFC types (a) the sm cells, (b) t cells and (c) the m cells, versus time (data is mean, n = 3). Black arrows show the times when feedings occurred under maximum flow rate.

According to the literature and best lab practices, this flow rate was sub-optimally low for maximum growth rate and concomitant maximum power output [39] however it was a practical limitation that could be overcome. Similar to our previous study [9], due to struvite precipitation which is probably enhanced under such low flow rates, the anodic liquid volume gradually reduced to 1 ml. For overcoming the low performance due to fuel unavailability, the cells were occasionally fed under maximum flow rate, until the anolyte volume was fully replaced (see arrows in Fig. 3).

All experiments were performed at room temperature (22 ± 2 °C). Voltage output for each MFC was individually recorded in volts (V) versus time by using an Agilent data logger (KEYSIGHT, 34972A LXI data acquisition/Switch) unit. The polarisation experiments were performed using a variable resistor (Centrad Boite A Decades De Resistances DR07). Data were produced by sweeping resistor values from 1MΩ to 3.74 Ω. The time interval between resistance changes was 3 min. All data presented are the mean of 3 triplicate MFCs.

## Results and discussion

### Power output behavior of the different MFC types

As can been seen in Fig. 3a, for the sm group type, the increase of the cathode surface area (sm4: s.a 22.86 cm^2^) improved the absolute power output when compared with the MFCs with a smaller cathode surface area (sm1, sm2, sm3: s.a 13.75 cm^2^). Moreover, the CV anode electrode (sm1) performed slightly better in comparison with the CC anodic electrode (sm3). The AC modification on the surface of the CV anode, presented the lower power performance for the sm group units (sm4>sm1>sm3>sm2)*.*

As can been in Fig. 3b, the AC modified anode (t3), presented the best power performance, followed by the CC anode (t2), the CV anode (t1) and the SS modified cathode (t4) for the t units (t3>t2>t1>t4). However, after 27 days of continuous operation, this behavior was shifted and the higher power output values originated from the SS cathode cells (t4), followed by t3, t1 and t2 group units (t4>t3>t1>t2). This behavior was consistent throughout the end of work.

Additionally, the results for the m group, revealed that the CC anode electrode (m3), exhibited very good power performance when compared with the AC anode (m2), the CV anode (m1) and the SS modified cathode (m4) (m3>m2>m1>m4) (Fig. 3c). Several “bad” data points originated after interventions to the experiment such as polarisation experiments (high values), have been removed from Fig. 3. Moreover, the power output sharp peaks result from replacing the anolyte with fresh urine by maximizing the flow rate of the pump. This intervention enhanced for short time the power of the units. In spite of this, power decreased during long term operation. This was attributed to the struvite precipitation due to low flow rate [8].

Among the different ceramic types, the best performing was the sm type (in the range of 60–100 μW), followed by the t type (in the range of 40–80 μW) and the m type (in the range of 20–40 μW). The same general trend was observed during long term operation. Specifically, the sm cells had higher durability in comparison with the t and the m units. Namely, the power output of the t cells gradually decreased after approximately 60 days of operation, while the power of the m cells decreased after approximately 20 days of continuous operation (with the exception of the m3 type). Moreover, the sm type showed a better performance stability through time (140 days) (Fig. 3).

### Polarisation experiments of the different MFC types during time

**Fig. 4:**
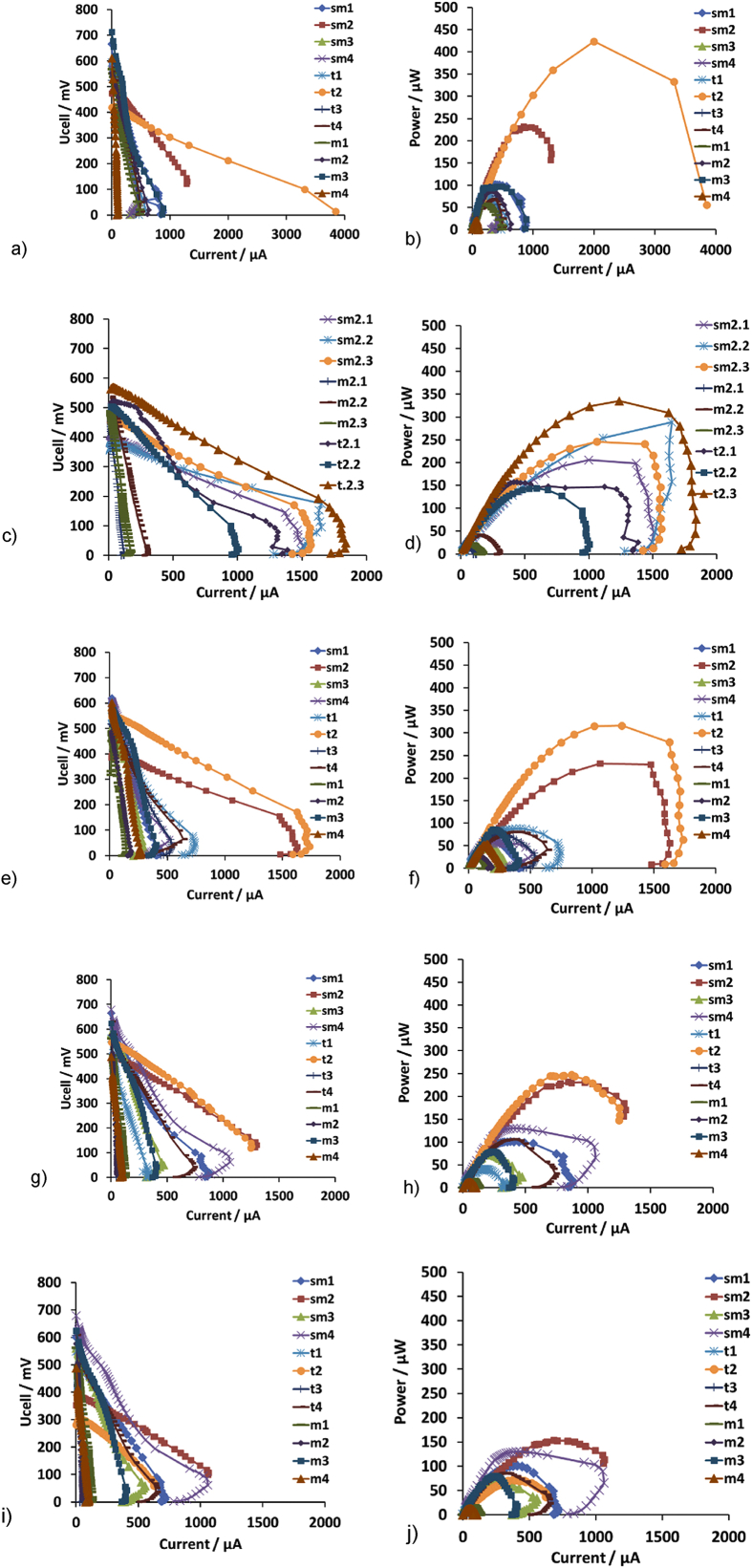
Cell voltage Ucell (a,c,e,g,i) and power output (b,d,f,h,j) versus current for the best performing cells for each group at days 7, 14, 21, 59 and 96, respectively. Fig. 4c and d, present the results for each unit of group 2 (sm2.1–2.3; m2.1–2.3; t2.1–2.3).

Five sets of polarisation experiments were conducted during the continuous operation of the units, at the following times: 1st at 7 days, 2nd at 14 days, 3rd at 21 days, 4th at 59 days and 5th at 96 days. Fig. 4 shows the voltage Ucell and power output versus current for the best performing cells for each group, during time. Fig. 4c and d, present the results for each unit of group 2.

The polarisation experiments showed that for the sm group, the higher values of maximum power Pmax, were achieved from the sm units with the AC modified anode (162 μW, 288 μW, 224 μW, 162 μW, 119 μW for the 1st, 2nd, 3rd, 4th and 5th polarisation experiment, respectively), followed by the group with the increased cathode surface area (130 μW, 65 μW, 130 μW, 116 μW for the 1st, 3rd, 4th and 5th polarisation experiment, respectively), the CV anode (101 μW, 70 μW, 101 μW, 38 μW for the 1st, 3rd, 4th and 5th polarisation experiment, respectively) and finally the CC anode (88 μW, 64 μW, 87 μW, 50 μW for the 1st, 3rd, 4th and 5th polarisation experiment, respectively) (sm2>sm4>sm1>sm3).

Furthermore, the polarisation experiments for the t group revealed that the higher values of maximum power Pmax, resulted from the AC modified anode (423 μW, 148 μW, 283 μW, 30 μW for the 1st, 2nd, 3rd, and 4th polarisation experiment, respectively), followed by the SS modified cathode (99 μW, 47 μW, 106 μW, 71 μW for the 1st, 3rd, 4th and 5th polarisation experiment, respectively), the CC anode (94 μW, 14 μW, 5 μW, 5 μW for the 1st, 3rd, 4th and 5th polarisation experiment, respectively) and the CV anode (78 μW, 65 μW, 22 μW, 9 μW for the 1st, 3rd, 4th and 5th polarisation experiment, respectively) (t2>t4≥t3>t1).

Additionally, the results for the m group, revealed that the higher values of maximum power Pmax resulted from the CC anode electrode (94 μW, 77 μW, 8 μW, 8 μW for the 1st, 3rd, 4th and 5th polarisation experiment, respectively) followed by the AC anode (72 μW, 24 μW, 12 μW, 8 μW and 8 μW for the 1st, 2nd, 3rd, 4th and 5th polarisation experiment, respectively), the CV anode (59 μW, 21 μW, 6 μW, 6 μW for the 1st, 3rd, 4th and 5th polarisation experiment, respectively) and the SS cathode (19 μW, 51 μW, 12 μW, 12 μW for the 1st, 3rd, 4th and 5th polarisation experiment, respectively) (m3>m2>m1>m4).

This behavior was consistent throughout the end of work, for all MFC types. Similarly, to the power behavior versus time (Fig. 3), the maximum power values decreased during long term operation with the sm group to exhibit the biggest durability. However, in contrast to the absolute power values, the higher values of maximum power output originated from the AC modified anode cells, followed by the SS modified cathode and the increased s.a. Cathode, for both the sm and the t cells. Among all groups the highest maximum power (423 μW) was achieved from the t2 type. The aforementioned results are in consistence with the work produced from You et al. [40], who also observed that the addition of a micro-porous layer (MPL) on the anodes, boosted the power performance. In particular, the maximum power output of the cells increased by 2.2 (304.3 mW) and 1.8 (253.9 mW) times compared to the power outputs produced from the plain CV and CC anodes respectively. Moreover, Gajda et al. [41] indicated that the activated carbon ink on the anode electrode (carbon veil) increased the power performance of the cell by 77% (37.9 mW). The activated carbon particles which were added on the carbon veil, boosted the electrocatalytic activity and increased the surface area of the anode. The same research group observed that the addition of micro-nanostructure activated carbon on the carbon veil, resulted to a 10-fold increase of the power performance (power output 3.7 mW) [42].

## Conclusions

The operation of 36 newly designed ceramic MFC units was assessed and compared using different or modified materials. The sm cells performed better and had better durability through time, when compared with the ceramic cylinders having bigger ID. Moreover, m units showed the lower performance. The results from polarisation experiments revealed that the increase of the cathodic surface area, the modified AC anode and the addition of SS to the cathodic electrode, enhanced the power performance for both the sm and the t cells. However, this was not the case for the m cells where the CC anodic electrode had the best performance. The highest maximum power output obtained was 423 μW and originated from the t units with the AC modified anode. These findings indicate that there is a lot of space for boosting power performance per cell, by making simple interventions either to the design either to the electrodes by improving their characteristics, with the addition of relatively cheap materials (such as AC and SS). The next question to answer is how the combination of the above modifications would contribute to the power performance.

## Declaration of competing interest

The authors declare that they have no known competing financial interests or personal relationships that could have appeared to influence the work reported in this paper.
